# Phase Separation Clustering of Poly Ubiquitin Cargos on Ternary Mixture Lipid Membranes by Synthetically Cross-Linked Ubiquitin Binder Peptides

**DOI:** 10.1021/acs.biochem.4c00483

**Published:** 2025-02-26

**Authors:** Soojung Kim, Kamsy K. Okafor, Rina Tabuchi, Cedric Briones, Il-Hyung Lee

**Affiliations:** † Department of Chemistry and Biochemistry, Montclair State University, Montclair, New Jersey 07043, United States; ‡ Department of Biology, Montclair State University, Montclair, New Jersey 07043, United States

## Abstract

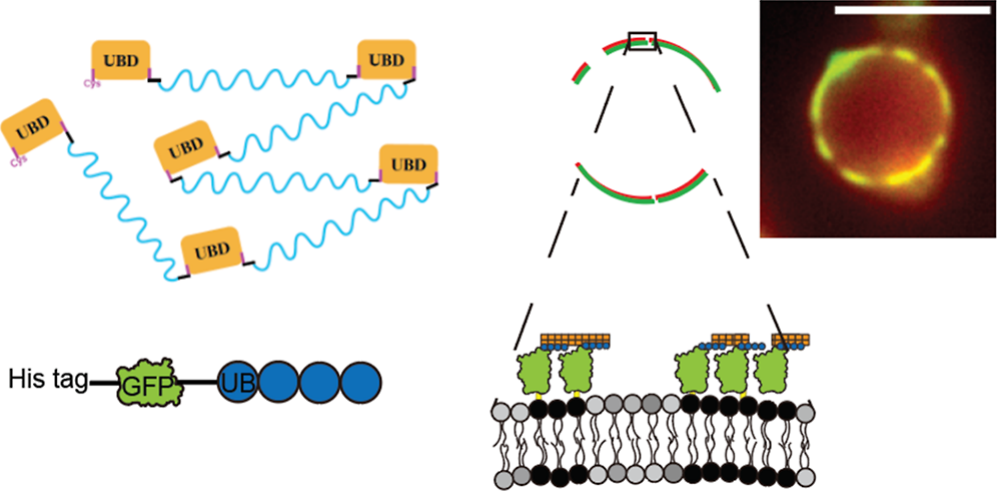

Ubiquitylation is involved in various physiological processes, such as signaling and vesicle trafficking, whereas ubiquitin (UB) is considered an important clinical target. The polymeric addition of UB enables cargo molecules to be recognized specifically by multivalent binding interactions with UB-binding proteins, which results in various downstream processes. Recently, protein condensate formation by ubiquitylated proteins has been reported in many independent UB processes, suggesting its potential role in governing the spatial organization of ubiquitylated cargo proteins. We created modular polymeric UB binding motifs and polymeric UB cargos by synthetic bioconjugation and protein purification. Giant unilamellar vesicles with lipid raft composition were prepared to reconstitute the polymeric UB cargo organization on the membranes. Fluorescence imaging was used to observe the outcome. The polymeric UB cargos clustered on the membranes by forming a phase separation codomain during the interaction with the multivalent UB-binding conjugate. This phase separation was valence-dependent and strongly correlated with its potent ability to form protein condensate droplets in solution. Multivalent UB binding interactions exhibited a general trend toward the formation of phase-separated condensates and the resulting condensates were either in a liquid-like or solid-like state depending on the conditions and interactions. This suggests that the polymeric UB cargos on the plasma and endosomal membranes may use codomain phase separation to assist in the clustering of UB cargos on the membranes for cargo sorting. Our findings also indicate that such phase behavior model systems can be created by a modular synthetic approach that can potentially be used to further engineer biomimetic interactions in vitro.

## 1 Introduction

Ubiquitylation is the enzymatic addition of the small protein ubiquitin (UB) to a target protein. As the name implies, ubiquitylation occurs in various physiological processes, such as signaling,^1^ DNA repair,^2^ and receptor trafficking.^3^ Because of its general and versatile role in the physiological processes of many organisms^4,5^ including humans, its biochemistry has been an intense area of study. For example, during endoplasmic reticulum-associated protein degradation (ERAD), misfolded proteins are ubiquitinated and destined for degradation.^6,7^ Maintaining such quality control systems is essential for cell survival. Cancerous cells are known to overburden the ERAD system; thus, ubiquitylation and its involvement in ERAD are of significant clinical interest as a therapeutic target to treat various cancers.^8^ During vesicle trafficking of plasma membrane cargos, proteins, such as clathrin^9^ and endosomal complex required for transport (ESCRT)^10,11^ family proteins, contain UB-binding domains that selectively bind to UB-bearing cargo for degradation or recycling of target membrane proteins.

Several groups recently reported the relevance of liquid–liquid phase separation or condensate formation during UB-mediated processes, particularly involving poly ubiquitin (polyUB), in which UB addition is repeated in a polymeric manner enabling specific recognition by multivalent binding partners.^12−19^ Multiple independent UB systems have been reported for their capability of forming protein condensates. These systems include Rad23B in proteasomes,^14^ ubiquitin in stress granules,^15,16^ p62 in selective autophagy,^17^ ESCRT-0 in vesicle trafficking,^18^ and NF-κB essential modulator (NEMO) in NF-κB signaling.^19^ This suggests that condensate formation is an inherent property of the UB system and plays an important role in UB-mediated processes. Protein condensates can act as membrane-less organelles by enriching a subset of proteins inside the condensates.^20,21^ This enrichment may enhance the kinetic rate of biochemical reactions catalyzing these processes.^22,23^ When condensate formation occurs on the lipid membranes containing proteins, it may form a cluster with these membrane proteins,^24,25^ which can enhance the kinetic rate of recruiting the downstream proteins involved in the processes.^26,27^ Therefore, understanding condensate formation involving UB is of great significance.

In the present study, we used a synthetic modular approach to create poly-UB and poly-UB-binding proteins via an artificial protein construct and synthetic peptide cross-linking. Wide-field fluorescence and difference interference contrast (DIC) microscopy were used to observe the outcome of condensate formation from various combinations of the UB proteins. We demonstrated that membrane protein cargos bearing the UB may cluster via phase separation. Previous reports have indicated that protein condensate formation and lipid raft formation may collaboratively form codomains on the membranes.^28−30^ We found that polyUB cargos can form similar codomains when tested with synthetic giant unilamellar vesicles (GUV) of ternary compositions, in which the three most common lipid types in the mammalian plasma membranes coexist (saturated chain phospholipids, unsaturated chain phospholipids, and cholesterol). This ternary composition is considered an excellent model system for studying lipid rafts or lipid domain formation behavior of the plasma membrane.^31^ We also found that such codomain formations are closely associated with the inherent property of polyUB proteins to form a phase-separated protein condensate that may be in its final state of fluidic or solid phase. This suggests that such valence-dependent cargo codomain formations may play an important role in the cargo clustering step of vesicle trafficking. A modular synthetic system may be used as a model system to study the physiological process systematically and to design biomimetic systems in vitro.

## 2 Methods

### 2.1 GUV Preparation

All lipids used were purchased from Avanti Polar Lipids, Inc. and stored in chloroform at −20 °C. GUVs were prepared by the gentle hydration method.^32,33^ The ternary mixture of GUVs consisted of a mixture of 1,2-dioleoyl-*sn*-glycero-3-phosphocholine (DOPC), 1,2-dipalmitoyl-*sn*-glycero-3-phosphocholine (DPPC), and cholesterol. For homogeneous GUVs, 42.5 mol % DOPC, 19.8 mol % DPPC, and 35.0 mol % cholesterol were used along with the functional lipids, including 0.2 mol % Texas Red-1,2-dihexadecanoyl-*sn*-glycero-3-phosphoethanolamine (TR-DHPE, Invitrogen) and 2.5 mol % nickel bound 1,2-dioleoyl-*sn*-glycero-3-[(*N*-(5-amino-1-carboxypentyl)iminodiacetic acid)succinyl] (Ni-DOGS). Next, 200–500 μg of lipid mixture was loaded into a round-bottomed glass flask and insufflated with high-purity nitrogen to generate a thin lipid film, which was subsequently desiccated in a vacuum chamber for at least 1 h at room temperature to remove residual chloroform. Lipid films were gently hydrated by the addition of 1 mL of 320 mM aqueous sucrose solution and incubation at 37 °C for 16–19 h. The GUVs were harvested by centrifugation at 12,000*g* for 5 min to remove aggregation and were stored at 4 °C for use within a day.

### 2.2 GUV-Protein Sample Preparation for Experimentation and Imaging

A circular cover glass (Thickness #1, Fisher Scientific) was immersed in a 1:1 mixture of clean water and isopropanol, then cleaned by bath sonication for 30 min followed by rinsing with ultrapure water. The coverglass was assembled into an Attofluor sample chamber (Invitrogen) with an O-ring to limit the total sample volume to 100–200 μL. The cover glass was surface passivated with 200 μL of 5 mg/mL bovine serum albumin (BSA) solution for 30 min. Residual BSA was rinsed and the buffer was exchanged with HEPES buffer solution. (20 mM HEPES, 150 mM NaCl, pH 7.4). All solutions were prepared with clean water that was reverse osmosed, filtered, and ion-exchanged multiple times using a Milli-Q filtration unit. (Millipore Sigma).

GUV solution (1–10 μL), to ensure a countable number of vesicles per image, was added to the chamber and allowed to equilibrate for 10 min. Representative z-stack images were captured at multiple unique positions to characterize the GUVs based on their shape, lamellarity, and size. To anchor the UB-green fluorescent protein (GFP) cargo to the membranes, His-tagged UB-GFP was added at a final concentration of ∼4 μM. At least 10 vol % of the current liquid volume was added to ensure homogeneous mixing. After a 30 min incubation, z-stack images were captured to monitor the successful binding of the cargo. Finally, UBD conjugate was added to cause an interaction with the UB cargos by adding ∼2 μM, concentration by UBD monomers, and incubating for 30 min. Many z-stack images were sampled as final state images. During the incubation steps, time-lapse images were captured to monitor the kinetic changes as needed. All experiments were performed at room temperature 22(±1)°C. Images were analyzed for the phase separation state of the individual vesicles that were well-defined as unilamellar vesicles.

### 2.3 Protein Condensate Formation

For solution-phase condensate formation, UB-GFP cargos (∼30 μM) and UBD conjugate (∼100 μM monomers) were mixed in an Eppendorf tube along with UBD monomer and incubated for at least 10 min at room temperature. The mixture was imaged directly by adding a drop of liquid on a clean cover glass assembled in the sample chamber. Fluorescence and DIC images were collected in the final state. Other mixtures included 200 μM flexible UB6 + 100 μM NEMOUBAN6 for the UB6/NEMOUBAN6 system and 70 μM UB6 + 120 μM UBA6 for the UB6/UBA6 system. For UB6/NEMOUBAN6 and UB6/UBA6, a fraction of the UB proteins were labeled with an organic fluorescent dye Sulfo-Cy5 (Lumiprobe) as the fluorescence signal.

### 2.4 Imaging Conditions

GUVs were imaged using a Nikon Ti2E-based inverted epifluorescence microscope system (Nikon, Japan) mounted on a vibration isolation table. The Nikon Apo 100× TIRF oil objective lens, with a numerical aperture of 1.49, was used with an sCMOS camera (Hamamatsu ORCA Flash 4.0, Hamamatsu, Japan) for image acquisition. An LED white light (Lumencor, Beaverton, OR), filtered by dichroic mirrors and optical filters to transmit selective wavelengths, was used to excite the GUV samples, which emitted green or red fluorescence signals for the GFP or Texas Red channels, respectively. Micromanager was used for the automatic acquisition of z-stack images from 1 μm–20 μm and the x and y positions were mechanically controlled to visualize the unique z-sections of the GUVs, which were analyzed by ImageJ software. For DIC imaging, the same scope was used with contrast polarization filters and bright field light illumination.^34^

### 2.5 Protein Purification and Peptide Synthesis

The UBD Conjugate was synthesized by cross-linking.^35−37^ The synthesis was carried out in a 2-step reaction. A synthetic cross-linker bearing maleimide (Mal) groups at both terminal ends linked by 3 repetitions of polyethylene glycol (PEG) was used to prepare 1,11-bismaleimido-triethylene glycol, which is abbreviated as BM-PEG3 (Thermo Fisher Scientific) First, UBD peptide produced to the purity of >99,9% (Genscript), was bound to the synthetic cross-linker using the Cys–Mal reaction overnight (>16 h) at room temperature by mixing 20 μL of 6.4 mM peptide monomer with 2 μL of 56.8 mM of the cross-linker (final 1x molar ratio) and 20 μL of 20 mM HEPES, pH 7.4, containing 150 mM NaCl. Next, a 10 mM final concentration of 2-mercaptoethanol was added to the reaction to quench the reactive functional groups for >30 min at room temperature. The UBD construct at this stage already had all the reported phase separation abilities, but we performed an additional cleanup procedure by dialyzing the protein overnight at 4 °C, with the same HEPES buffer as above. A 1 kDa molecular weight threshold dialysis membrane was used (G-Biosciences). The concentration of the peptide remaining was ∼400 μM as determined by Nanodrop UV absorption. (Thermo Fisher) Synthesis of other UBD conjugates, including a poly-l-lysine (PLL) backbone-based construct can be found in the Supporting Information (Appendix 1).

The sequence of the ESCRT-0 UBD peptide was as follows, which was originally a UB-interacting motif from STAM1A of the ESCRT-0 complex.^38,39^

**GCSKEEEDLA KAIELSLKEQ RQQGGSW**

Tryptophan and Cysteine Were Added for Spectroscopy Characterization and Efficient Cross-Linking.

For the purification of the modularly designed proteins, recombinant plasmids (Genscript) were transformed into competent *Escherichia coli* for overexpression.^30,32,40,41^ The strains included BL21AI (Invitrogen) and BL21(DE3), in which overexpression could be induced with 0.5 mM IPTG and arabinose (only for BL21AI). Flexible 6UB and 6UBA were expressed with BL21(DE3) and the other proteins were expressed in BL21AI. *E. coli* was grown at 37 °C and the proteins were overexpressed overnight at a lower temperature of 18 °C. The cells were harvested by centrifugation and the pellets were resuspended in HEPES buffer (20 mM HEPES, pH 7.4, 150 mM NaCl) and disrupted using a high-pressure French press (Glen Mills, Clifton, NJ). Proteins were separated from *E. coli* debris by centrifugation and purified using Ni-NTA affinity chromatography in a gravity column. Further purification was achieved by automated column chromatography (ÄKTA explorer, GH Healthcare) typically using a size exclusion chromatography of HiLoad Superdex75 or a Superdex200 column. The purified proteins were characterized by SDS-PAGE and spectroscopy and stored at −20 °C. Protein sequence information can be found in Appendix 2 of the Supporting Information.

For Cys-maleimide-labeling of the fluorescent probes (Sulfo-Cy5-malemide, Lumiprobe) to the purified proteins, ∼1–4-fold excess concentration of the maleimide dye was added to either the final protein product or to the sample during the purification process before performing size exclusion and incubated at 4 °C overnight (>16 h) followed by removal of the dyes by desalting column or dialysis. The typical labeling ratio estimated by nanodrop spectroscopy was 15%.

## 3 Results and Discussion

### 3.1 Purification and Synthesis of Modular Ubiquitin Proteins

**Figure 1:**
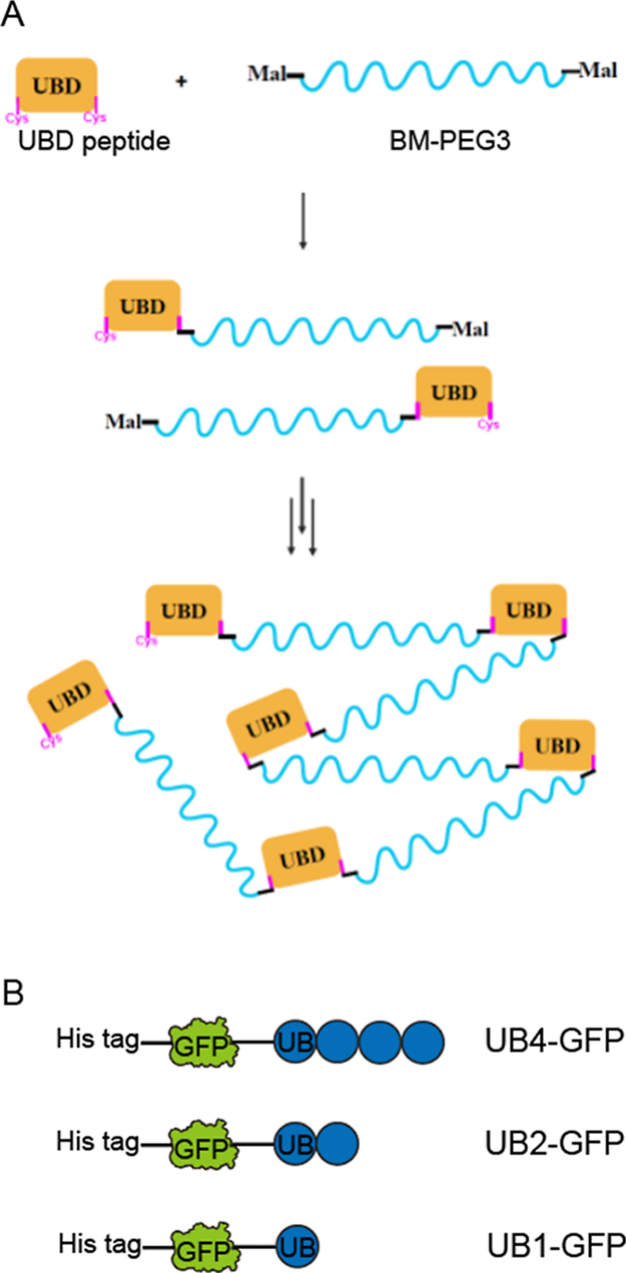
Modular UB proteins. (A) The synthetic UBD conjugate was created by cross-linking multiple UBD peptides with the synthetic cross-linker, BM-PEG3. The cysteine–maleimide reaction was used for synthetic cross-linking. (B) PolyUB membrane cargos were created by purifying GFP-bearing linear polyUB with varying lengths.

To test the ability of the membrane UB cargos to cluster by phase separation, a synthetic multivalent UB-binding conjugate was designed and prepared (Figure 1A) Briefly, the UBD domain taken from the ESCRT-0 sequence, one of the UB binding motifs in the protein complex,^39^ was synthesized as a monomer peptide bearing Cys residues for the Cys–Mal reaction. A synthetic cross-linker containing maleimides at both ends was attached to the peptide using one of the two Cys residues on the peptide. This peptide-cross-linker naturally polymerized into larger constructs through repeated Cys-maleimide reactions. The reaction was quenched by 2-mercaptoethanol and any unreacted monomers were removed by dialysis. Based on the characterization by size exclusion chromatography, it was estimated that a typical multivalent conjugate that participated in the reaction contained a median of 9 UBD peptides per conjugate and spanned a molecular weight of 10–40 kDa when synthesized under the conditions that we used (Appendix 2 of the Supporting Information). Using this bioconjugate approach, we tested the multivalent interaction of interest at its purest form as a model system that may be modified for further studies. In this way, we are not limited by purification by mammalian cell expression^42^ of every component involved in the interaction, which is often required to study a specific UB interaction system. This may serve as a stepping stone toward the synthesis of a biomimetic system using the polyUB interaction in vitro. Various polyUB constructs were also designed as model membrane cargos with ubiquitylation (Figure 1B). GFP served the model cargo proteins, while providing the necessary fluorescence signal for microscopy. *E. coli* expressed the protein, which was purified by a series of chromatography steps. The His-tag was used for affinity purification and was preserved for membrane binding by His-Ni chelation with Ni-DGS lipids.

### 3.2 PolyUB Membrane Protein Cargo Phase Separates into Lipid–Protein Codomain on the Ternary Mixture GUVs

**Figure 2:**
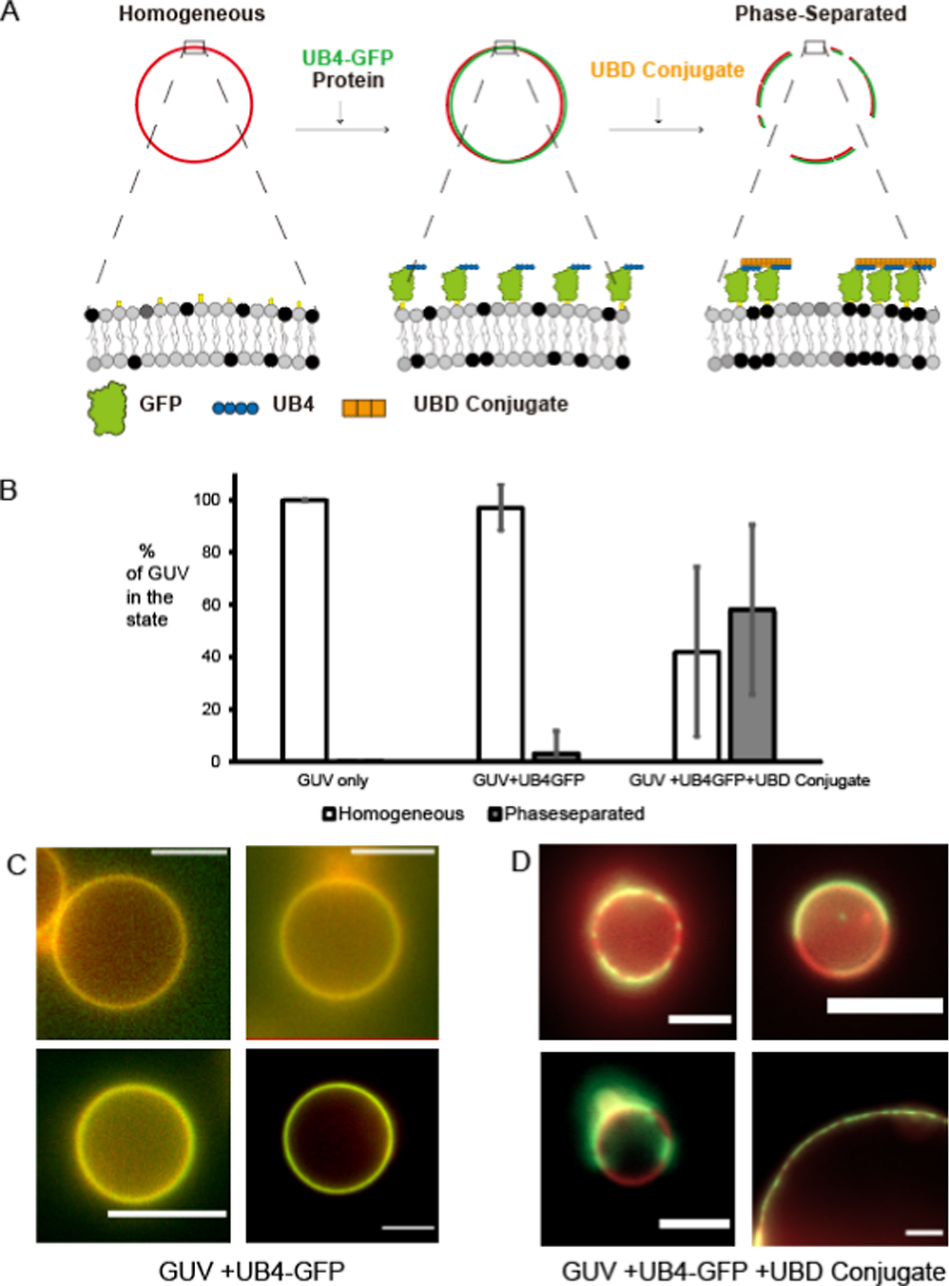
Phase separation of the polyUB cargo on the membranes by the polyUB-UBD conjugate interaction. (A) Schematic of the lipid–protein interaction transitioning from homogeneous to phase-separated after introducing UB4-GFP and the UBD conjugate. The lipid composition was DOPC (42.5%), DPPC (19.8%), cholesterol (35%), Ni-DGS (2.5%), and TR-DHPE (0.2%0), and the reaction included 4 μM UB4-GFP and ∼2 μM UBD conjugate (based on the monomer concentration). Gray and black colored lipids indicate the saturated and unsaturated chain lipids (DPPC and DOPC). Lipid domains still contain all lipids in the original composition; however, the ratio of the lipid composition is different between the inside and outside of the domains.^43^ Other lipids were omitted for brevity. (B) Statistical distribution of the resulting phase behavior of the GUVs. Statistical analysis from more than 20 image stacks captured from 3 independent experiments. Error bars represent standard deviations among all the image stacks analyzed. Each image was analyzed for the percentage of vesicles in each phase state and the numbers were averaged for the outcome. The number of vesicles analyzed was *N* > 125 for each state. (C) Example images of GFP cargo fluorescence (green) overlapped with Texas Red lipid fluorescence (red), maintaining homogeneous behavior after introducing the UB4-GFP protein. (D) Example images showing phase-separated behavior after introducing the UBD Conjugate. Scale bars are 5 μm.

To examine phase separation during polyUB membrane cargo clustering using the modular synthetic proteins, we reconstituted the UB4-GFP cargo on the lipid membranes mimicking the composition of the physiological plasma membranes where saturated chain lipids, unsaturated chain lipids, and cholesterol coexist.^31,44^ GUVs with a ternary mixture composition including DOPC, DPPC, and cholesterol were created (DOPC 42.5 mol %, DPPC 19.8 mol %, Cholesterol 35.0 mol %, Ni-DGS 2.5 mol %, and TR-DHPE 0.2 mol %). Next, 2.5 mol % of what was supposed to be DOPC, when only the ratio of the ternary mixture was considered, was replaced into a functional headgroup lipid Ni-DGS, which was used to permanently anchor the UB4-GFP cargo to the lipid membranes (see Supporting Information Figure S2 for negative control data). TR-DHPE (0.2 mol %) was introduced as a fluorescent reporter for the lipids. The GUVs were first introduced into the sample chamber and subjected to fluorescence imaging. The UB4-GFP cargos were then bound to the membranes, and finally, the UBD conjugate was introduced to cause a binding interaction between UB4-GFP and the UBD conjugate (Figure 2A). Each step was examined by multicolor fluorescence imaging to sample many vesicles. The resulting phase states of the vesicles were statistically analyzed at each stage of incubation.

Ternary mixture lipids are known to separate into liquid-ordered (lo) and liquid-disordered (ld) binary lipid domains under specific conditions.^31,45−48^ In the present study, the GUVs were predominantly homogeneous at room temperature and this distribution did not change after UB4-GFP cargo was introduced on the membranes. However, once the UBD conjugate was added, it caused approximately 60% of the vesicles, on average, to form phase-separated cargo domains (Figure 2B). Example images of the homogeneously distributed and phase-separated cargos are also shown in Figure 2C. As shown in the examples, UB4-GFP cargo clustering into the cargo-enriched space leaves the rest of the cargo-depleted space readily visible only after introducing the UBD Conjugate. This is because the multivalent interaction between UB4-GFP and the UBD conjugate caused the cargos to phase separate and form a liquid condensate on the membranes.^49^

Previous studies suggested that the driving force of the ternary mixture of lipid membranes to form the lo, ld domains and the driving force of the proteins on the membranes to form protein condensate domains can collaboratively cause the formation of codomains, in which the protein domains and lipid domains align in space.^28−30,32^ Our results suggest that this principle applies to the polyUB cargos on the plasma membrane and mimics ternary mixture lipid membranes as well. This is important because it suggests that the polyUB cargos on the membranes are under a condition in which they can readily cluster into the phase domain to aid downstream processes, such as vesicle trafficking. This suggests that the plasma membrane and endosomal cargo sorting step is assisted by the composition of the membranes to better organize the cargo proteins on the membranes in space.

### 3.3 Co-Domain Formation is Valency Dependent

After observing the ability of the polyUB cargos on the membranes to form a codomain by its interaction with the multivalent binder UBD conjugate, we systematically examined the valency dependence of the phase separation. Favorable multivalent interactions are often the key to promoting protein condensates by shifting the equilibrium toward the formation of a dense condensate structure.^40,41^ In physiological processes, ubiquitylation occurs at various lengths and morphologies;^50,51^ thus, the dependence on valency is important.

**Figure 3:**
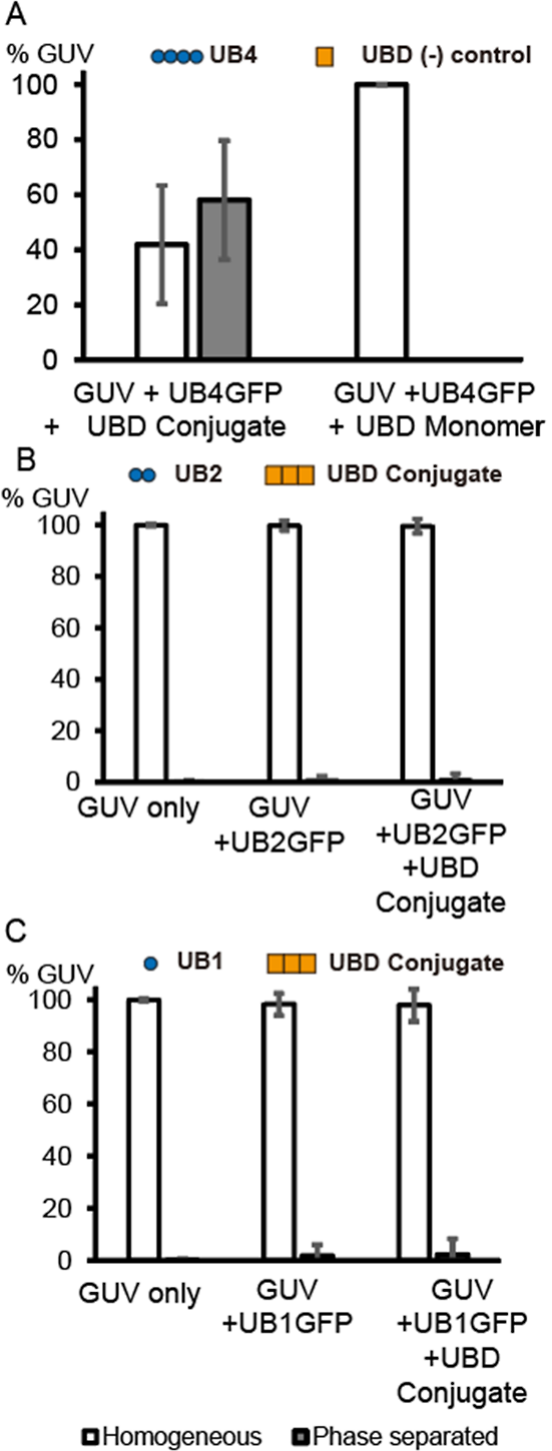
Statistical analysis of the phase separation state of the cargo vesicles. (A) UB4-GFP + UBD monomer negative control experiment compared with the UB4-GFP + UBD conjugate experiment from Figure 2B. (B) UB2-GFP and UBD conjugate experiment. (C) UB1-GFP and UBD conjugate experiment. The lipid compositions and protein concentrations were identical to those in Figure 2. DOPC 42.5%, DPPC 19.8%, cholesterol 35%, Ni-DGS 2.5%, and TR-DHPE 0.2%, and incubation conditions of the proteins, 4 μM UB4-GFP and ∼17 μM UBD Conjugate (based on the monomer concentration). The only exception is the control experiment A, in which we used DOPC 37.5% and Ni-DGS 10%. Error bars represent standard deviations from each image. Statistical distribution of the resulting phase behavior of the GUVs. Statistical analysis from more than 20 image stacks captured from 3 independent experiments. Error bars represent standard deviations within the images analyzed. Each image was analyzed for the percentage of vesicles in each phase state, and the numbers were averaged for the outcome. The number of vesicles analyzed was *N* > 125 for each state of incubation.

First, a negative control experiment was performed with a monomer UBD binder. Identical concentrations of the monomer UBD binder peptides were introduced during the last step of the experiment, instead of the UBD conjugate. A similar ternary GUV composition and UB4-GFP concentration were used as shown in the original experiment of Figure 2, rather than the use of the UBD monomer. In contrast to the original result with the UBD conjugate (Figure 3A), the UBD monomer resulted in practically no change in its introduction to the system (Figure 3B). There are identical numbers of UBD motifs in the solution; thus, it is the multivalency in the binding interaction that caused the difference between codomain formation versus no significant change at all.

**Figure 4:**
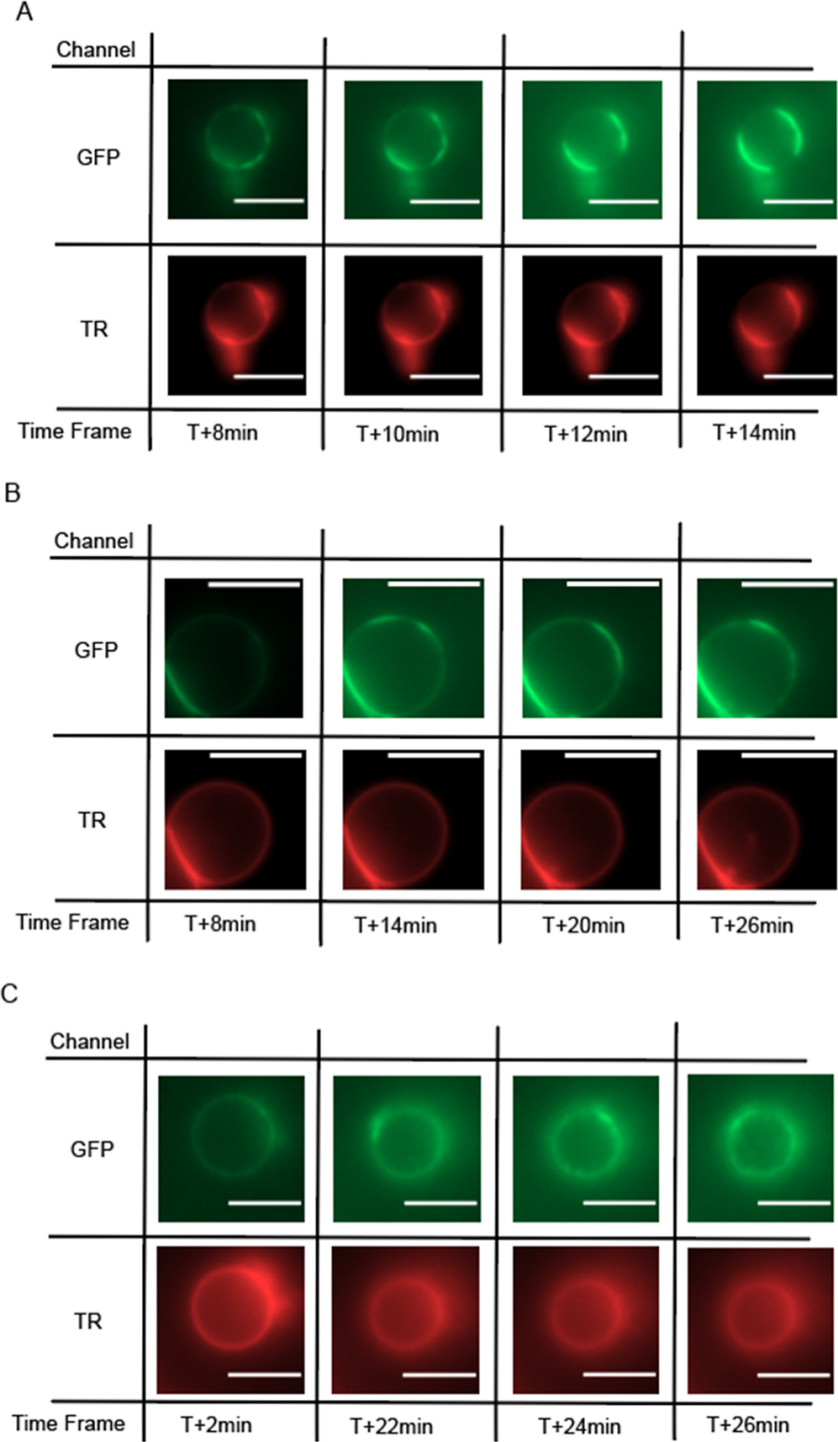
Time-lapse images showing the dynamic changes of the phase separation states. Time zero is defined as the time the UBD conjugate was added to the sample with UB4-GFP cargo on ternary mixture GUVs. (A) An example time-lapse captured at T + 8, 10, 12, and 14 min. What was originally homogeneous became clustered at T + 8 min, then coarse into phase-separated domains toward T + 14 min. The TR signal shows that lipids follow the localization of the protein cargo. (B) An example time-lapse taken at T + 8, 14, 20, and 36 min. Similarly, the originally homogeneous vesicle developed clustering regions as a result of phase separation. In this case, colocalization of the lipid channel was not evident. (C) Another time-lapse example. The focus moved up a bit toward the later time point, which clearly shows the phase separation of the upper top portion of the vesicle. Lipid and protein signals tend to have some correlation, but clear overlapping between two channels was not always evident to the extent the intensity signal could be resolved. Scale bars are 5 μm.

Next, we performed a similar series of experiments by varying the length of ubiquitylation of the membrane cargos. The lipid composition and protein concentrations used were identical to those in the Figure 2 experiment with UB4-GFP. The UBD conjugate was kept constant as a multivalent binder, while the length of polyUB by 2 and 1 was varied. As shown in Figure 4B,C, shorter UB cargos exhibited less of a tendency toward phase separation, as evidenced by less than 5% in the final statistics of the phase-separated cargo vesicles. This is consistent with general expectations, in which greater multivalency promotes the formation of phase-separated protein condensates, and polymeric ubiquitylation has a distinct ability to form such codomains on the membranes compared with mono- or diubiquitylation of the cargos. This suggests a way to affect the fate of membrane protein cargos through poly ubiquitylation.

### 3.4 Dynamic Changes in the Time-Lapse Images Showing Collaborative Domain Forming Interaction between Lipids and Proteins

**Figure 5:**
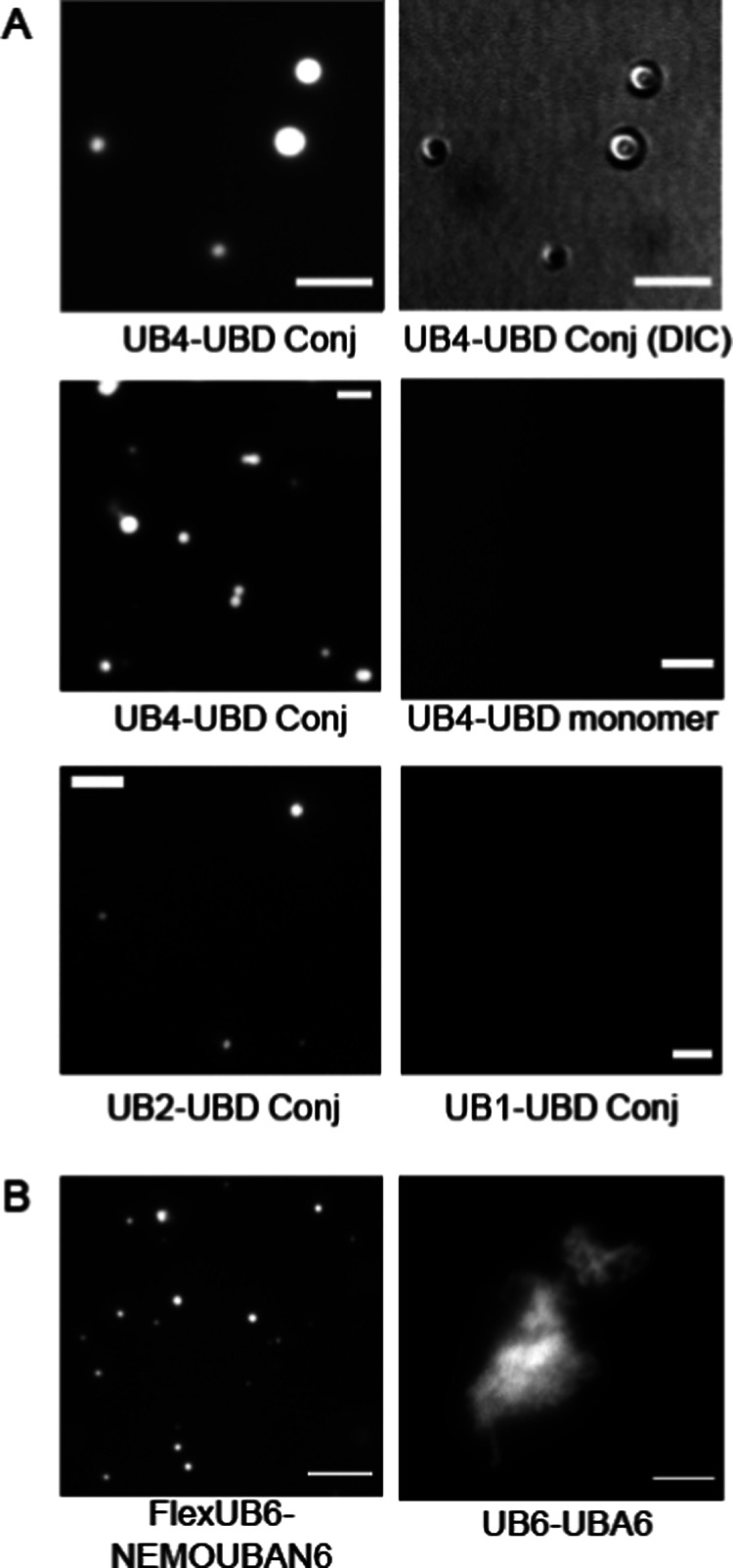
Protein condensate formation of UB proteins in solution. (A) Example fluorescent condensate images between UB-GFP of varying lengths (UB4, UB2, and UB1) and the UBD conjugate and the UBD monomer. The fluorescence and DIC images in the first row are the matching images. (B) Example condensate images formed between other polyUB and UB-binding proteins. FlexUB6 and NEMOUBA6 form liquid-like droplets, whereas UB6 and UBA6 form solid-like structures. Scale bars are 5 μm.

To obtain the kinetic trace during the statistical change of the phase separation state, we performed a time-lapse imaging analysis for the UB4-GFP and UBD conjugate interaction. Multicolor images were captured every 2 min after the initial introduction of the UBD conjugate to the sample. Time zero was defined as the exact time the UBD conjugate was introduced. Figure 5 shows three time-lapse examples of the typical change from the homogeneous to the phase-separated, or cargo clustered state after the incubation began. As evidenced by the GFP channel images, which represent the spatial distribution of the UB4-GFP cargos on the membranes, the initial homogeneous fluorescence distribution starts developing bright and dark spots across the membranes, which indicate binary separation into the phase domains. The separation domain may show the behavior of fluctuation and coarsening in the time scale of minutes, as they are fluidic domains, but eventually maintain a separated state. When the TR channel images were compared, which represent the behavior of the lipids,^52^ colocalization of the two images was not always evident. For example, in Figure 4A, it is evident that lipid separation spatially overlaps with protein cargo separation as the bright/dark regions in the TR channels reproduce the bright/dark regions in the GFP channels. However, in Figure 4B,C, the lipid signal appears homogeneous, while clustering in the protein channel is visible, at least within the limitation that the clustering is resolvable. Questions remain as to what constitutes the condition in which coclustering in proteins and lipids becomes obvious. It is worth noting that lipids and protein cargos may move independently in a hypothetical scenario, in which proteins and lipids do not interact strongly. It is the formation of a codomain between lipids and proteins by a favorable intermolecular interaction that forces them to move correlated with space. We can at least argue that cargo protein and lipid clustering are closely correlated, as there was no observed case in which lipids and proteins in each phase separated in an uncorrelated manner. Further systematic study of lipid composition dependence may shed light on the matter.

### 3.5 PolyUB Proteins Have the Potential to Form Protein Condensates in Solution at Various States and Their Ability is Strongly Correlated with Codomain Formation on the Membranes

Recent studies have shown the ability of UB proteins to form protein condensate in solution.^14−16,18,19,53^ We evaluated protein condensate formation in solution without the membranes at higher concentrations than that of the membrane experiments (>30 μM each). UB cargo proteins and UBD conjugate were mixed in solution, provided sufficient time to interact (>10 min) at room temperature, and observed by microscopy. As shown in Figure 5A, the UB4-GFP and UBD conjugates readily formed many condensates with round droplet shapes >1 μm in diameter. The droplets were enriched with UB4-GFP as evidenced by the bright fluorescence signal, and the droplets were visible by DIC imaging. UB2-GFP, when tested for the same interactions, formed some visible droplets, albeit smaller and fewer in number. The enrichment of UB2-GFP cargos inside the droplets was several-fold lower compared with the UB4-GFP as estimated by fluorescence intensity (Supporting Information, Figure S5). UB1-GFP with no multivalency did not form droplets. This suggests that UB4-GFP, with its high multivalency, had the greatest capacity to form a protein condensate and enrich it with the most cargo molecules. This correlated with the statistical result of the most phase-separated cargo vesicles formed on the membranes (Figure 3).

A general caution should be taken when discussing the relevance of solution protein condensate on the lipid membranes because such model rests on the premise that protein behavior remains unchanged between solution and membrane environments. In our case, we argue that there is obvious and strong correlation between the solution condensate formation and the collaborative cargo clustering on the membranes, but solution phase condensate may not necessarily guarantee the similar condensate formation on the lipid membranes.

We also tested several other UB and UB-binding proteins with respect to their ability to form a protein condensate in solution. Figure 5B shows example images of the solution phase behavior of other UB proteins (see Supporting Information Figure S6 for schematics of the proteins). Flexible-UB6 is labeled by an organic fluorescence dye, which is a construct of polyUB linked to a flexible GGS spacer between UBs. When incubated with another modular protein of NEMOUBAN6, containing six repeats of the UBAN domain from the NEMO proteins, formed round condensate droplets comparable with the ones formed between the UB4-GFP and UBD conjugate. This system used a different UB binding domain and the purification of the modular construct instead of peptide synthesis. This shows that the modular repetition of UB and UB-binding multivalency can reproduce condensate formation behavior, regardless of the exact nature and structure of the multivalent binding interactions.

It is important to note that not all modular interactions involving multivalent interactions between polyUB and UB-binding proteins will result in a liquid-like phase separation.^54,55^ PolyUB proteins may form a condensate in a solid-like state, which occurred between UB6 (linear polyUB with 6 UB repeats labeled with an organic dye) and UBA6 (linear 6 UBA domain^56^ repeats overexpressed and purified in *E. coli*). Solid-like structures tend to have irregular shapes instead of round shapes, and they tend to grow into gigantic rock-like spiky structures greater than 10 μm in length (Figure 5B). In the field of phase separation of protein condensates, it is known that in a certain subregion of the phase space, the proteins may form solid-like structures.^54,55,57−59^ Its relevance to the physiological interaction of polyUB cargos remains to be elucidated; however, they have the ability to form a solid-like structure under certain conditions.

The condensate droplets formed between UB4-GFP and the UBD conjugate showed a fluidic or liquid-like behavior that was experimentally verified. The droplets readily recovered fluorescence after the photobleaching experiment (Supporting Information Figure S4A), suggesting that they exist in a dynamic exchange of molecules with the solution phase, a property of the liquid-like phase.^60^ Multiple droplets often coalesced into larger single droplets with round edges that nonspecific solid aggregates are unable to do^61^ (Supporting Information Figure S4B,C). This suggests that the condensates formed between UB4-GFP and the UBD conjugate are fluidic, liquid-like droplets and not nonspecific solid aggregates or polymeric solid structures.

### 3.6 Observed Phase Behavior between the Polymeric UB Cargos and the Synthetic UBD Constructs is Universal: PLL-UBD Construct Results

A similar trend of valence-dependent phase separation cargo clustering was observed when tested with another synthetic construct based on the PLL backbone, namely, the PLL-UBD construct. We observed coclustering on the homogeneous ternary mixture membranes, valence dependence, and a correlation with the droplet-forming ability in solution. Despite the matching trend, there was a concern that the positive charge of the PLL backbone affected the phase behavior in a somewhat unpredictable manner. Cationic interaction is known to modulate the domain phase behavior.^62^ PLL specifically is capable of penetrating the membranes of negatively charged lipids^63^ and PLL may cause morphology changes to the membranes.^64^ Although we were certain that a similar coseparation was occurring based on the valence-dependent trend, we could not completely decouple the effect of valence and charges. Therefore, we decided to provide this in the Supporting Information and retained the data from the UBD conjugate (BM-PEG3 based) in the main text for brevity (Supporting Information Appendix S3 and Figures S7–S11). However, this suggests that our synthetic approach and resulting phase behavior are generally observable in similarly created systems. PLL is a commonly used peptide; thus, the strategy of using it as a backbone to attach multiple binding motifs opens the possibility of creating various synthetic systems^37,65,66^ to test multivalent binding interactions.

## 4 Conclusion

In this study, we demonstrated that polyUB cargos on ternary mixture lipid membranes that mimic the composition of the plasma membranes can collaboratively cluster into phase-separated domains that are dependent on the multivalent interaction with UB-binding proteins. We constructed a simple model system to test this using a modularly designed synthetic UBD conjugate and polyUB cargos at varying lengths. Membrane clustering is closely correlated to their ability to form a protein condensate in solution. PolyUB proteins in multivalent interactions have a general ability to form a condensate, but the resulting state of the condensate may be liquid-like or solid-like depending on the conditions and the proteins involved.

The model system based on modular synthesis can test interactions independent of the effect of the remaining protein structure and is easily expandable to test fundamental intermolecular interactions that are important for the desired outcome. It may be applied to design in vitro biomimetic systems using condensate-forming interactions.^67,68^ However, it should be noted that the synthetic approach is missing potential allosteric effects of the physiological proteins that may be important to finely regulate each specific physiological interaction.^39,69^ As we observed, our UBD conjugate was relatively small in size, potentially altering the steric effect of the physiological proteins; thus, the result should be interpreted within the limitation of the model system.

Even considering the limitation of the model system approach, it is evident that polyUB cargos on membranes can cluster by forming phase-separated codomains assisted by lipid rafts. This suggests that poly ubiquitylation may be used to promote the spatial sorting of cargos on the plasma and endosomal membranes during vesicle trafficking processes. Multivalent binding and polyubiquitination are very common in UB systems; thus, their synergetic interaction with lipid membranes deserves attention in the related processes. The ESCRT-0 protein complex, from which the UBD domain was taken, has multiple UB binding sites that formed a condensate in yeast vacuoles.^18^ Many downstream ESCRT family proteins also contain UB binding motifs while capable of forming polymeric structures on the membranes for the purpose of remodeling the membranes.^10,70^

In our previous study using SUMO cargo proteins, we postulated that when the collaborative codomain formation was in play, phase separation tended to promote domain formation, while there exists an opposing force of steric pressure involving high-density cargo on the membranes.^32,71^ These two opposing forces compete to determine the final statistical distribution of cargo domain separation. For SUMO cargo proteins, the high-density cargo condition caused by the 10 mol % Ni-DGS functionalized lipids incubated with comparable concentrations of SUMO3-GFP caused the resulting cargo distribution to be exclusively homogeneous, whereas lower Ni-DGS concentrations of 5 and 1 mol % reduced the steric pressure to increase the distribution of phase separation.^32^ Reversing steric pressure is known to be protein size-dependent.^71^ We observed some preliminary examples in which phase-separated UB4-GFP cargos were reversed into a homogeneous distribution as a result of the binding interaction at high cargo density, which we could observe for the UBD conjugate and also another poly UB binding construct (Figure S3). However, we did not have enough statistical confidence to include the results here. One of our future projects is to study the lipid composition dependence in depth.

Considering the increasing number of case studies on polyUB-based condensates, it is evident that such condensate formation plays a role in physiological processes. We learned that UB and UB-binding proteins form not only a liquid-like condensate, but also a solid-like structure in a reproducible manner. There have been no systematic studies regarding what leads to the formation of a solid-like structure instead of a fluidic condensate; therefore, systematic studies of the phase diagram of UB proteins will be a future area of study. Our working hypothesis is that highly dense multivalent molecules interact in space beyond a certain binding affinity, causing solid-like structure formation by “a bit too tight” packing of the proteins that require further scrutiny in the future.

## Supporting Information Available

Supporting Information including Appendix 1, 2, 3 that provide additional information regarding characterization of the UBD Conjugate, full sequence of the proteins used, Synthesis of the PLL-UBD Conjugate and Figures S1–S11 that provide additional figures of peptide characterization, control imaging experiments, solution condensate fluidity test, quantification of the condensate fluorescence intensity, schematics of the modular proteins used, all experimental data of PLL UBD Conjugate (PDF)

## Abbreviations

BM-PEG3: 1,11-bismaleimido-triethylene glycol
DIC: differential interference contrast microscopy
DOPC: 1,2-dioleoyl-*sn*-glycero-3-phosphocholine
DPPC: 1,2-dipalmitoyl-*sn*-glycero-3-phosphocholine
ESCRT: endosomal sorting complex required for transport
GFP: green fluorescence protein
GUV: giant unilamellar vesicle
Mal: maleimide
NEMO: NF-kappa-B essential modulator
NHS: *N*-hydroxysuccinimide
Ni-DGS: nickel bound 1,2-dioleoyl-*sn*-glycero-3-[(*N*-(5-amino-1-carboxypentyl)iminodiacetic acid)succinyl]
PEG: poly ethylene glycol
PLL: poly-l-lysine
polyUB: poly ubiquitin
TCEP: tris(2-carboxyethyl)phosphine
TR-DHPE: texas red-1,2-dihexadecanoyl-*sn*-glycero-3-phosphoethanolamine
UB: ubiquitin
UBA: ubiquitin association domain
UBAN: ubiquitin-binding domain in ABINs and NEMO
UBD: ubiquitin binding domain
