# Supplementary material for: Phase Separation Clustering of Poly Ubiquitin Cargos on Ternary Mixture Lipid Membranes by Synthetically Cross-Linked Ubiquitin Binder Peptides
Source: Biochemistry. 2025 Feb 26;64(6):1212–21. doi: 10.1021/acs.biochem.4c00483 (PMC11924212; doi:10.1021/acs.biochem.4c00483)
Supplement: Supplementary file 1 — bi4c00483_si_001.pdf [file bi4c00483_si_001.pdf]

Supporting Information for the article: “Phase Separation Clustering of Poly Ubiquitin Cargos on Ternary Mixture Lipid Membranes by Synthetically Cross-Linked Ubiquitin Binder Peptides.”

Soojung Kim[1†], Kamsy K. Okafor[2†], Rina Tabuchi[1], Cedric Briones[1],

and Il-Hyung Lee[1*]

Affiliations: [1]Department of Chemistry and Biochemistry, Montclair State University, Montclair, NJ 07043, USA, [2]Department of Biology, Montclair State University, Montclair, NJ 07043, USA

† **These authors contributed equally** (co-first authors)

***Correspondence to** Il-Hyung Lee, leei@montclair.edu

The file includes:

Appendix 1. Characterization of the synthetic UBD Conjugate

Appendix 2. Full sequence of the proteins used

Appendix 3. Synthesis, characterization, and phase separation clustering by PLL backbone based UBD Conjugate (PLL-UBD Conjugate)

S1-S11. Supplementary Figures

Appendix 1. Characterization of the synthetic UBD Conjugate

We performed experiments to characterize the synthesized UBD Conjugate. Since our synthesis relies on stochastic events of UBD peptides being crosslinked by the bivalent crosslinker BM-PEG3, the resulting UBD Conjugate is a mixture of n-mers that can be characterized by the average number of UBD peptides bound per construct. Figure S1 shows the result of the characterization experiment by the size exclusion chromatography.

To learn the size distribution of the final UBD Conjugates, the sample was run by the Superdex S75 column by automated chromatography system using neutral hepes buffer. When compared to the standard calibration run by known molecular weight standards, it was estimated that the resulting UBD Conjugate has molecular weight distribution of 10-40kD where 30kD would be the median molecular weight.

Wen can reasonably assume that the monomer unit would be one UBD peptide + one croslinker. Molecular weight of this monomer unit is 3476 D. ~30kD median size means about 9-mer being most abundant construct. Since each peptide bears two cysteines (one near N-terminal, one at C-terminal) and each polymer bears two maleimide groups, we can expect only linear polymerization (it cannot in theory branch in a way the size exponentially grows). This measurement provides us some reasonable estimation as to how many repetition of the binding domain the construct has in average. This is important as evidenced by the valency dependence of the clustering interaction of the UB cargos on the membranes.

Appendix 2. Full sequence of the proteins used

Uniprot ID annotated

UBD peptide (from STAM1, Q92783)

GCSKEEEDLA KAIELSLKEQ RQQGGSWC

UB4-GFP (UB from F5GZ39)

MGMQIFVKTL TGKTITLEVE PSDTIENVKA KIQDKEGIPP DQQRLIFAGK QLEDGRTLSD
YNIQKESTLH LVLRLRGGMQ IFVKTLTGKT ITLEVEPSDT IENVKAKIQD KEGIPPDQQR
LIFAGKQLED GRTLSDYNIQ KESTLHLVLR LRGGMQIFVK TLTGKTITLE VEPSDTIENV
KAKIQDKEGI PPDQQRLIFA GKQLEDGRTL SDYNIQKEST LHLVLRLRGG MQIFVKTLTG
KTITLEVEPS DTIENVKAKI QDKEGIPPDQ QRLIFAGKQL EDGRTLSDYN IQKESTLHLV
LRLRGGCLEV LFQGPMVSKG EELFTGVVPI LVELDGDVNG HKFSVSGEGE GDATYGKLTL
KFICTTGKLP VPWPTLVTTL TYGVQCFSRY PDHMKQHDFF KSAMPEGYVQ ERTIFFKDDG
NYKTRAEVKF EGDTLVNRIE LKGIDFKEDG NILGHKLEYN YNSHNVYIMA DKQKNGIKVN
FKIRHNIEDG SVQLADHYQQ NTPIGDGPVL LPDNHYLSTQ SKLSKDTNEK RDHMVLLEFV
TAAGITLGMD ELYKENLYFQ GLEHHHHHH

UB2-GFP

MGMQIFVKTL TGKTITLEVE PSDTIENVKA KIQDKEGIPP DQQRLIFAGK QLEDGRTLSD
YNIQKESTLH LVLRLRGGMQ IFVKTLTGKT ITLEVEPSDT IENVKAKIQD KEGIPPDQQR
LIFAGKQLED GRTLSDYNIQ KESTLHLVLR LRGGCLEVLF QGPMVSKGEE LFTGVVPILV
ELDGDVNGHK FSVSGEGEGD ATYGKLTLKF ICTTGKLPVP WPTLVTTLTY GVQCFSRYPD
HMKQHDFFKS AMPEGYVQER TIFFKDDGNY KTRAEVKFEG DTLVNRIELK GIDFKEDGNI
LGHKLEYNYN SHNVYIMADK QKNGIKVNFK IRHNIEDGSV QLADHYQQNT PIGDGPVLLP
DNHYLSTQSK LSKDTNEKRD HMVLLEFVTA AGITLGMDEL YKENLYFQGL EHHHHHH

UB-GFP

MQIFVKTLTG KTITLEVEPS DTIENVKAKI QDKEGIPPDQ QRLIFAGKQL EDGRTLSDYN
IQKESTLHLV LRLRGGCLEV LFQGPMVSKG EELFTGVVPI LVELDGDVNG HKFSVSGEGE
GDATYGKLTL KFICTTGKLP VPWPTLVTTL TYGVQCFSRY PDHMKQHDFF KSAMPEGYVQ

ERTIFFKDDG NYKTRAEVKF EGDTLVNRIE LKGIDFKEDG NILGHKLEYN YNSHNVYIMA
DKQKNGIKVN FKIRHNIEDG SVQLADHYQQ NTPIGDGPVL LPDNHYLSTQ SKLSKDTNEK
RDHMVLLEFV TAAGITLGMD ELYKENLYFQ GHHHHHHHH

UB6

MQIFVKTLTG KTITLEVEPS DTIENVKAKI QDKEGIPPDQ QRLIFAGKQL EDGRTLSDYN
IQKESTLHLV LRLRGGMQIF VKTLTGKTIT LEVEPSDTIE NVKAKIQDKE GIPPDQQRLI
FAGKQLEDGR TLSDYNIQKE STLHLVLRLR GGMQIFVKTL TGKTITLEVE PSDTIENVKA
KIQDKEGIPP DQQRLIFAGK QLEDGRTLSD YNIQKESTLH LVLRLRGGMQ IFVKTLTGKT
ITLEVEPSDT IENVKAKIQD KEGIPPDQQR LIFAGKQLED GRTLSDYNIQ KESTLHLVLR
LRGGMQIFVK TLTGKTITLE VEPSDTIENV KAKIQDKEGI PPDQQRLIFA GKQLEDGRTL
SDYNIQKEST LHLVLRLRGG MQIFVKTLTG KTITLEVEPS DTIENVKAKI QDKEGIPPDQ
QRLIFAGKQL EDGRTLSDYN IQKESTLHLV LRLRGGMQIF VKTLTGKTIT LEVEPSDTIE
NVKAKIQDKE GIPPDQQRLI FAGKQLEDGR TLSDYNIQKE STLHLVLRLR GGMQIFVKTL
TGKTITLEVE PSDTIENVKA KIQDKEGIPP DQQRLIFAGK QLEDGRTLSD YNIQKESTLH
LVLRLRGGMQ IFVKTLTGKT ITLEVEPSDT IENVKAKIQD KEGIPPDQQR LIFAGKQLED
GRTLSDYNIQ KESTLHLVLR LRGGMCENLY FQGHHHHHHH H

FlexUB6

MQIFVKTLTG KTITLEVEPS DTIENVKAKI QDKEGIPPDQ QRLIFAGKQL EDGRTLSDYN
IQKESTLHLV LRLRGGSGGS GGSGGSGGMQ IFVKTLTGKT ITLEVEPSDT IENVKAKIQD
KEGIPPDQQR LIFAGKQLED GRTLSDYNIQ KESTLHLVLR LRGGSGGSGG SGGSGGMQIF
VKTLTGKTIT LEVEPSDTIE NVKAKIQDKE GIPPDQQRLI FAGKQLEDGR TLSDYNIQKE
STLHLVLRLR GGSGGSGGSG GSGGMQIFVK TLTGKTITLE VEPSDTIENV KAKIQDKEGI
PPDQQRLIFA GKQLEDGRTL SDYNIQKEST LHLVLRLRGG SGGSGGSGGS GGMQIFVKTL
TGKTITLEVE PSDTIENVKA KIQDKEGIPP DQQRLIFAGK QLEDGRTLSD YNIQKESTLH
LVLRLRGGSG GSGGSGGSGG MQIFVKTLTG KTITLEVEPS DTIENVKAKI QDKEGIPPDQ
QRLIFAGKQL EDGRTLSDYN IQKESTLHLV LRLRGGSGGS GGSGGSGGWC ENLYFQGHHH
HHHHH

NEMOUBAN6 (from NEMO, Q9Y6K9)

MHHHHHHHHE NLYFQGCWGG SMQLEDLKQQ LQQAEEALVA KQEVIDKLKE EAEQHKIVME

TVPVLKAQAD IYKADFQAER QAREKLAEKK ELLQEQLEQL QREYSKLKAS SQESGGSGGS

GGSGGSMQLE DLKQQLQQAE EALVAKQEVI DKLKEEAEQH KIVMETVPVL KAQADIYKAD

FQAERQAREK LAEKKELLQE QLEQLQREYS KLKASSQESG GSGGSGGSGG SMQLEDLKQQ

LQQAEEALVA KQEVIDKLKE EAEQHKIVME TVPVLKAQAD IYKADFQAER QAREKLAEKK

ELLQEQLEQL QREYSKLKAS SQESGGSGGS GGSGGSMQLE DLKQQLQQAE EALVAKQEVI

DKLKEEAEQH KIVMETVPVL KAQADIYKAD FQAERQAREK LAEKKELLQE QLEQLQREYS

KLKASSQESG GSGGSGGSGG SMQLEDLKQQ LQQAEEALVA KQEVIDKLKE EAEQHKIVME

TVPVLKAQAD IYKADFQAER QAREKLAEKK ELLQEQLEQL QREYSKLKAS SQESGGSGGS

GGSGGSMQLE DLKQQLQQAE EALVAKQEVI DKLKEEAEQH KIVMETVPVL KAQADIYKAD

FQAERQAREK LAEKKELLQE QLEQLQREYS KLKASSQES

UBA6 (from Ubiquilin, Q9UMX0)

MQNPEVRFQQ QLEQLSAMGF LNREANLQAL IATGGDINAA IERLLGSGGS GGSGGSGGSQ

NPEVRFQQQL EQLSAMGFLN REANLQALIA TGGDINAAIE RLLGSGGSGG SGGSGGSQNP

EVRFQQQLEQ LSAMGFLNRE ANLQALIATG GDINAAIERL LGSGGSGGSG GSGGSQNPEV

RFQQQLEQLS AMGFLNREAN LQALIATGGD INAAIERLLG SGGSGGSGGS GGSQNPEVRF

QQQLEQLSAM GFLNREANLQ ALIATGGDIN AAIERLLGSG GSGGSGGSGG SQNPEVRFQQ

QLEQLSAMGF LNREANLQAL IATGGDINAA IERLLGSGGS GGSGGSGGSW CENLYFQGHH

HHHHHH

Appendix 3. Synthesis, characterization, and phase separation clustering by PLL backbone based UBD Conjugate (PLL-UBD Conjugate)

PLL-UBD Conjugate: UBD domain taken from the ESCRT-0 sequence, one of the UB binding motifs in the protein complex, was synthesized as a monomer peptide bearing Cys residues for Cys-Mal reaction. (Figure S7) A synthetic crosslinker bearing Maleimide and NHS ester on each end was attached to the peptide first. This peptide-crosslinker was lastly attached to the PLL by Lys-NHS ester reaction. Multiple UBD peptides could bind to each PLL as every Lys was open for the crosslinking reaction, thus the reaction was limited by the number of reactable peptide-crosslinkers. Based on the series of characterization by size exclusion chromatography and UV absorption spectroscopy, it was estimated that typical multivalent conjugate that participated in the reaction bears median 4 UBD peptides per conjugate spanning the molecular weight of 10-40kD when synthesized with the condition we used. (Figure S8)

PLL-UBD Conjugate Synthesis: Poly-L-Lysine backbone based construct or PLL-UBD Conjugate synthesis was carried out in a 3-step reaction. Synthetic crosslinker bearing Maleimide (Mal) group and N-Hydroxysuccinimide (NHS) ester in each end linked by 6 repetitions of poly ethylene glycol (PEG) was used. (BroadPharm, CA) Firstly, UBD peptide, produced to the purity of >99,9% (Genscript) was bound to the synthetic crosslinker using Cys-Mal reaction overnight by mixing 100µL of 1.0 mM peptide monomer with 100µL of 2.0 mM of the crosslinker (2x molar ratio) under the pH 7.4 20mM Hepes, 150mM NaCl, 2.5mM tris(2-carboxyethyl)phosphine (TCEP) condition at 4°C for 16–19 hours. Then the successfully crosslinked UBA peptides were bound to the PLL backbone by NHS ester-Lysine reaction overnight by adding with 25 µL of 4mg/mL Poly-L-Lysine (PLL, 4-20kD, MP Biomedicals) solution to the 0.44 mg/mL final concentration to incubate at 4°C for 16–19 hours. Finally, 5-10mM final concentration of 2-mercaptoethanol was added into the reaction to quench the reactive functional groups and stored at 4°C until used.

The sequence of the ESCRT-0 UBD peptide used was following, originally UB interacting motif from STAM1A of the ESCRT-0 complex.

GCSKEEEDLA KAIELSLKEQ RQQGGSW

Tryptophan and cysteine were added for spectroscopy characterization and efficient crosslinking.

Characterization of the synthetic PLL-UBD Conjugate: We performed experiments to characterize the synthesized PLL-UBD Conjugate. Since our synthesis relies on stochastic events of UBD peptides being crosslinked to the PLL backbone, the resulting UBD Conjugate is a mixture of n-mers that can be characterized by the average number of UBD peptides bound per backbone. Figure S8 shows the result of the characterization experiment including UV absorption spectroscopy (Nanodrop, ThermoFisher Scientific), size exclusion chromatography.

Firstly, we started by estimating the total amount of UBD peptide that successfully ended up binding to the backbone with molecular weight greater than 10kD. We introduced a tryptophan to the peptide sequence for UV absorption, thus UBD peptides selectively absorbed 280nm UV light while PLL and synthetic crosslinkers were invisible to UV absorption, which could be used as an advantage to selectively quantify the amount of UBD peptides. We compared the 280nm absorption of the UBD Conjugate as it is, and also after centrifugation with 10kD threshold Amikon tube (EMD Millipore) to selectively retain UB Conjugate that is >10kD after 20 fold

dilution. 280nm absorption was reduced to 10% of the original absorption before the filtration. This is not due to the change of concentration as the sample volume was kept constant. It suggests that about 90% of the UBA peptides fail to participate in the crosslinking reaction to make final molecular weight greater than 10kD. From this, we can reasonably assume that the majority of peptides do not end up being crosslinked to the backbone and they mostly remain as monomers. Thus the contribution of two cysteine residues causing crosslinking of multiple PLL backbone, although is a technical possibility, should be minimal.

Since even two UBD peptides being crosslinked to a 5kD PLL will have a molecular weight greater than 10kD, it can be assumed that any UBD Conjugate with meaningful multivalency that participated in the phase separation reaction should have molecular weight greater than 10kD. To learn the size distribution of the final UBD Conjugates, the sample was run by the Superdex S75 column by automated chromatography system using neutral hepes buffer. When compared to the standard calibration run by known molecular weight standards, it was estimated that the resulting UBD Conjugate has molecular weight distribution of 10-40kD where 25kD would be the median molecular weight.

The PLL we used for synthesis originally had molecule weight distribution of 4-20kD based on the manufacturer's characterization. If we assume 10kD of PLL backbone molecular weight, 10-40kD implies 0-8 mers per backbone with 4-mer being average for 25kD. (molecular weight of a peptide + synthetic crosslinker is 3.654kD)

UB cargo interaction of the PLL-UBD Conjugate: as explained in the main text, this variation of the modular synthetic construct showed a very similar outcome when interacted with polyUB cargos on the membranes. A series of supplementing figures S7-S11 show matching results studied by PLL-UBD interacting with UB cargos. Please be aware the exact lipid composition studied slightly differs from the experiments done with the original UBD Construct that are detailed in each figure legend. In case of PLL-UBD conjugate, we learned that highly concentrated positive charge of PLL backbone does affect the outcome of phase separation clustering by the unidentified mode of interaction. We could not unambiguously decouple this charge effect from the contribution of the phase separation, does PLL-UBD Conjugate result should be interpreted with caution.

S1. Supplementary figure 1

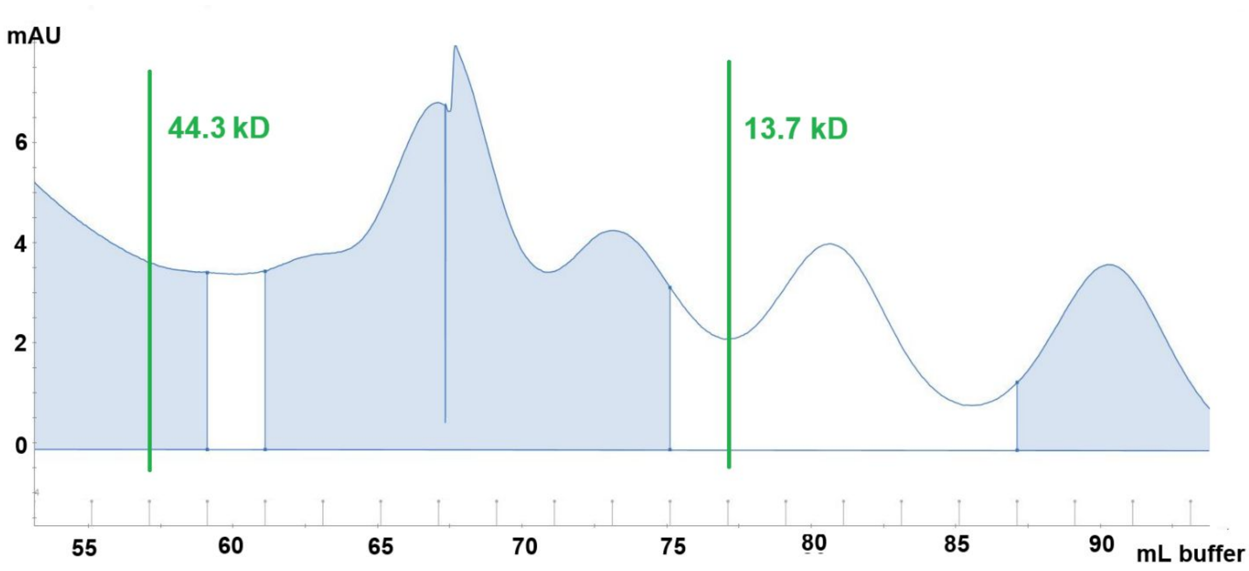

Figure S1. Molecular weight characterization of the UBD Conjugate (based on BM-PEG3 crosslinking). Superdex75 automated size exclusion analysis of the UBD conjugates shows the size is distributed around 10-40 kD range with median peak to be about ~30 kD. Earlier peak largely overlaps with the expected void peak location. Slight discontinuity of the mid peak is due to brief stop and continuation of the chromatography run which did not affect the size distribution analysis.

S2. Supplementary figure 2

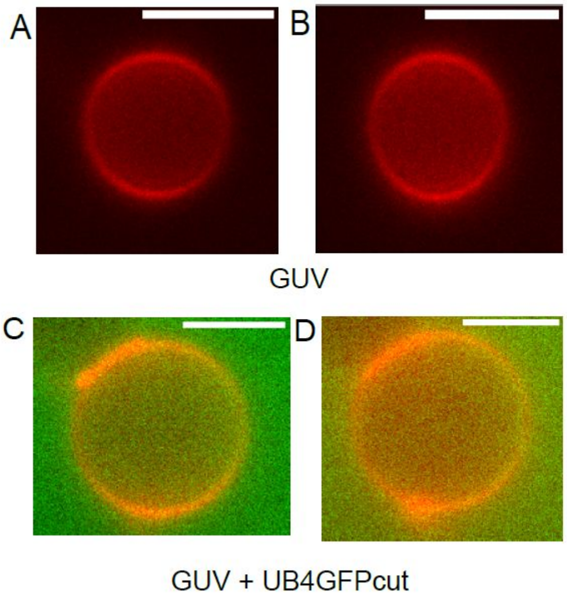

Figure S2. Negative control of UB4GFP_cut interaction with GUVs. (A, B) Example images of homogenous GUVs in Texas Red lipid fluorescence (red) channel. (C, D) Example images of unbound UB4-GFPcut cargo, which is a UB4-GFP construct where his-tag was removed by TEV digestion and affinity chromatography. Background fluorescence (green) shows with no colocalization with Texas Red lipid fluorescence (red), representative of unbound protein. The composition of the GUVs used for this control was DOPC 25%, DPPC 39.8%, cholesterol 25%, Ni-DGS 10%, and TR-DHPE 0.2%. It proves that the proteins are not capable of binding to the membranes without the Ni-His tag interaction.

S3. Supplementary figure 3

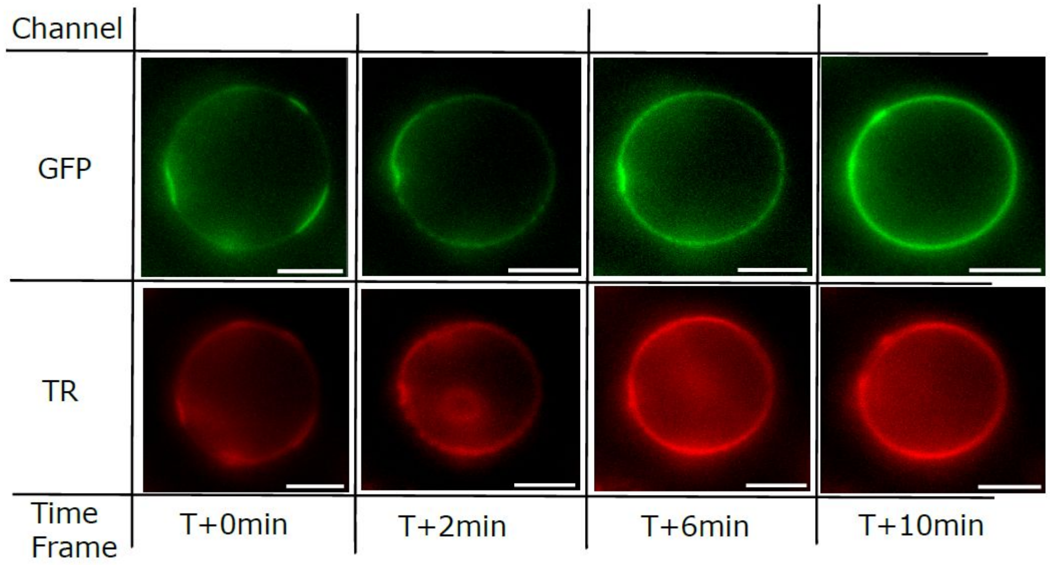

Figure S3. Time lapse images show dynamic changes of the phase separation states in Texas Red and GFP channel of a GUV and UB4GFP and UBA6. Time lapse taken at T+0min, 2min, 6min, and 10min. Images show an example kinetic trace reversal of phase separated GUVs becoming homogenous after the addition of the UBA6 at time zero. The composition of the GUVs used for this control was DOPC 25%, DPPC 39.8%, cholesterol 25%, Ni-DGS 10%, and TR-DHPE 0.2%. This experiment was performed by preparing the GUVs, then incubating with UB4-GFP (4 μM) as we did for the experiment in Figure 2. Finally UBA6 (>10μM) was added to start taking the time lapse movie.

S4. Supplementary figure 4

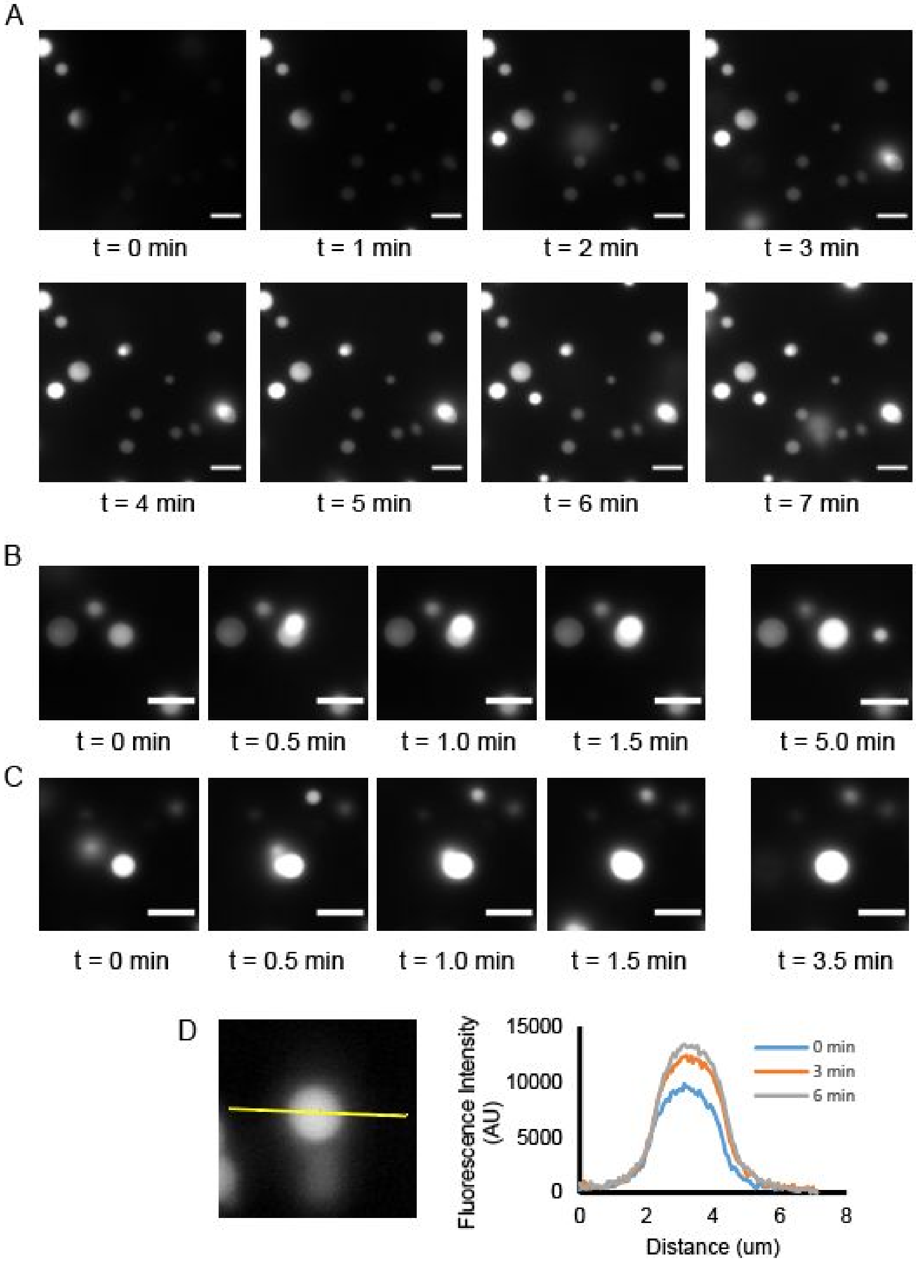

Figure S4. Liquid-like property of the protein droplets formed between UB4-GFP and UBD Conjugate. (A) Fluorescence recovery after photobleaching experiments on the droplets. GFP fluorescence images were taken. Condensates at the middle of the images were photobleached as a square pattern. As time elapses, their fluorescence intensities recover by fluidic exchange of molecules in solution. A partially bleached condensate recovery is also visible on the side. All images within the time-lapse series were scaled identically for the brightness and contrast. (B) Example of condensate droplets coalescing. Multiple droplets in the process of merging is evident. (C) Another example of condensate droplets coalescing. Coalescing behavior suggests the condensates are fluidic and liquid-like by nature. (D) Intensity profile plot of an example phase separated droplet FRAP across the yellow line. Background intensity was subtracted for clarity.

S5. Supplementary figure 5

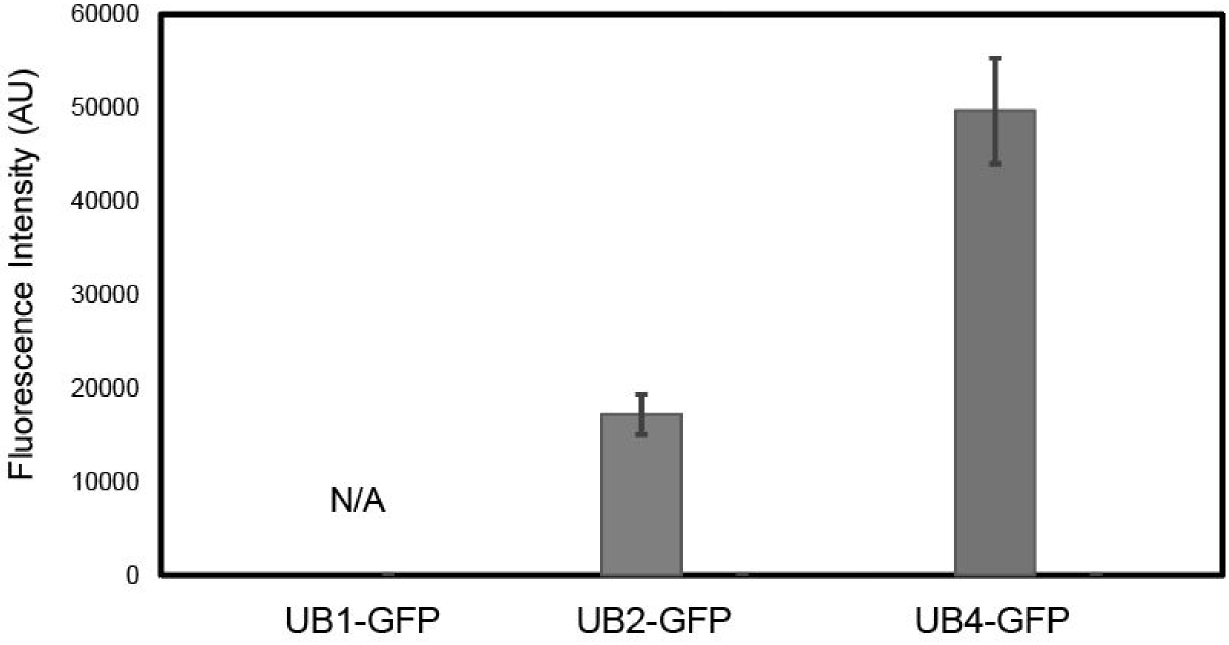

Figure S5. Peak fluorescence intensity at the middle of each droplet was quantified for comparison. Error bars represent standard deviations of droplet intensity values of at least 50 unique droplets from 3 z-stack images for each UB cargo protein. It is clear that longer UB chain-based droplets are more enriched with the cargo fluorescence. UB1-GFP cargo in combination of the UBD Construct did not have enough number of droplets to quantify the intensity unambiguously.

S6. Supplementary figure 6

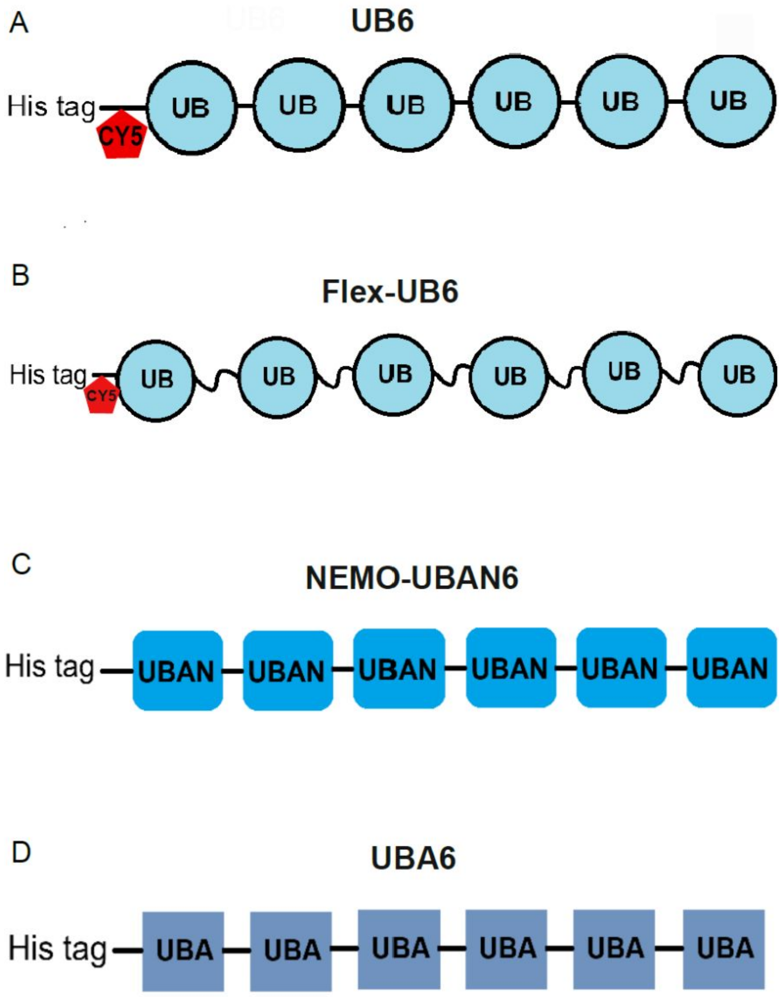

Figure S6. Schematics of other modular UB proteins. (A) UB6, polyUB membrane cargos were created by purifying His-tagged repetition of six linear polyUB with a cysteine residue for Cy5 dye attachment. (B) FlexUB6, polyUB membrane cargos were created by purifying His-tagged repetition of six linear polyUB with flexible -GGS- linkers and a cysteine residue for Cy5 dye attachment. (C) NEMOUBA6, Six linear UBAN domains. (D) UBA6, Six linear UBA domains from Ubiquilin.

S7. Supplementary figure 7

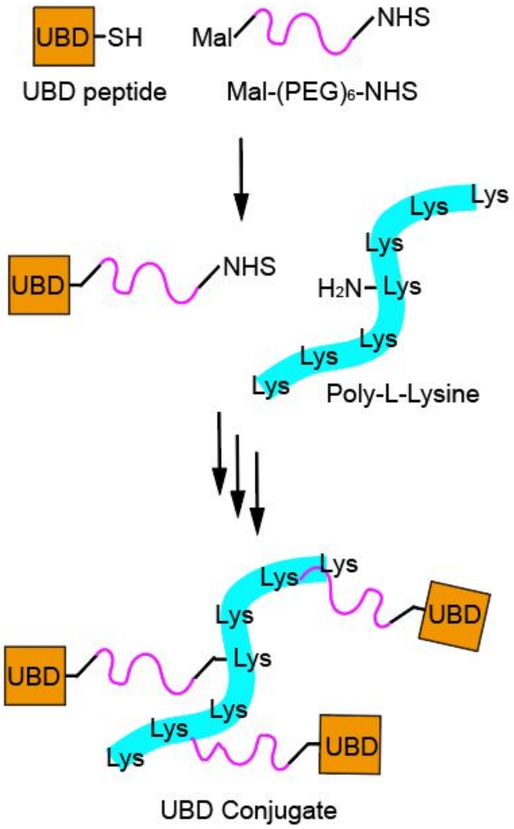

Figure 7. Modular synthesis of the PLL based UBD conjugate. PLL-UBD conjugate was created by crosslinking multiple UBD peptides into a PLL by synthetic crosslinkers.

S8. Supplementary figure 8

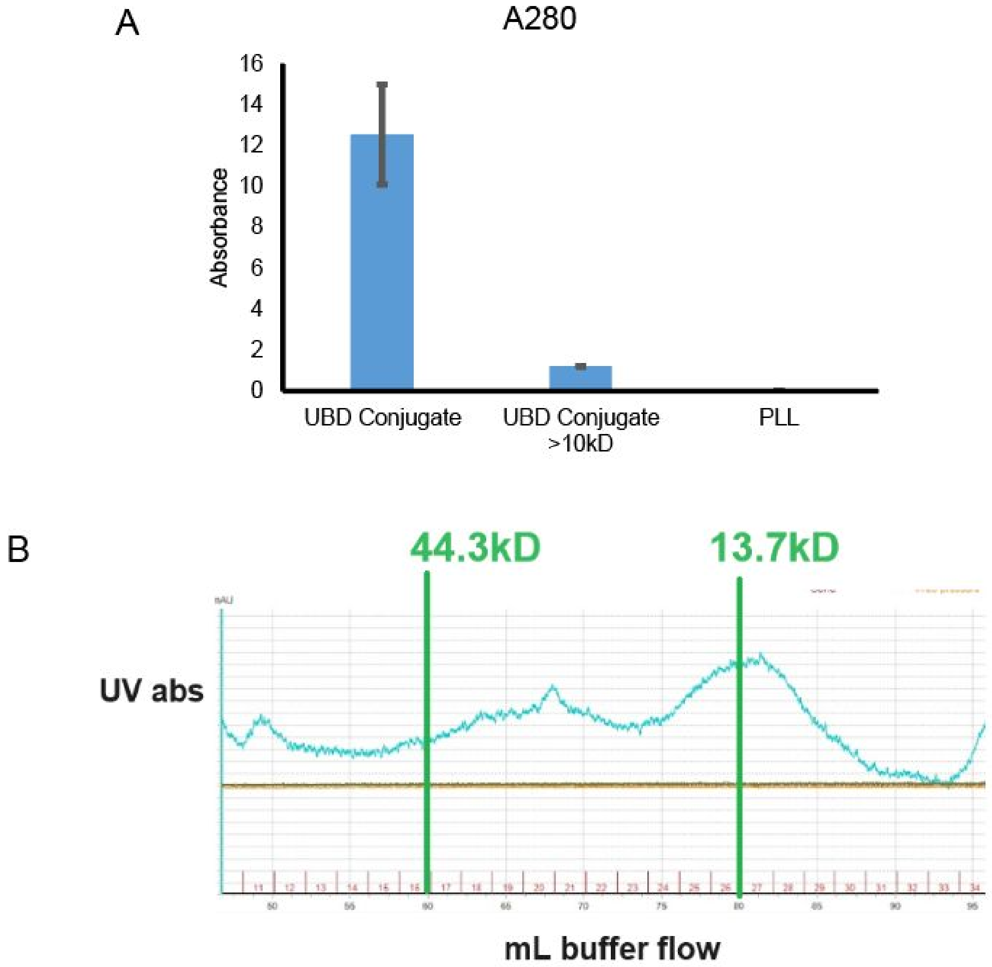

Figure 8. Molecular weight characterization of the PLL backbone based UBD Conjugate. (A) Nanodrop UV absorption shows that PLL has no UV absorption at all and about 10% of the original UBD peptide end up participating in the crosslinking reaction to make final molecuarweight > 10kD. (B) Superdex75 automated size exclusion analysis of the UBD conjugates shows the size is distributed around 10-40kD range.

S9. Supplementary figure 9

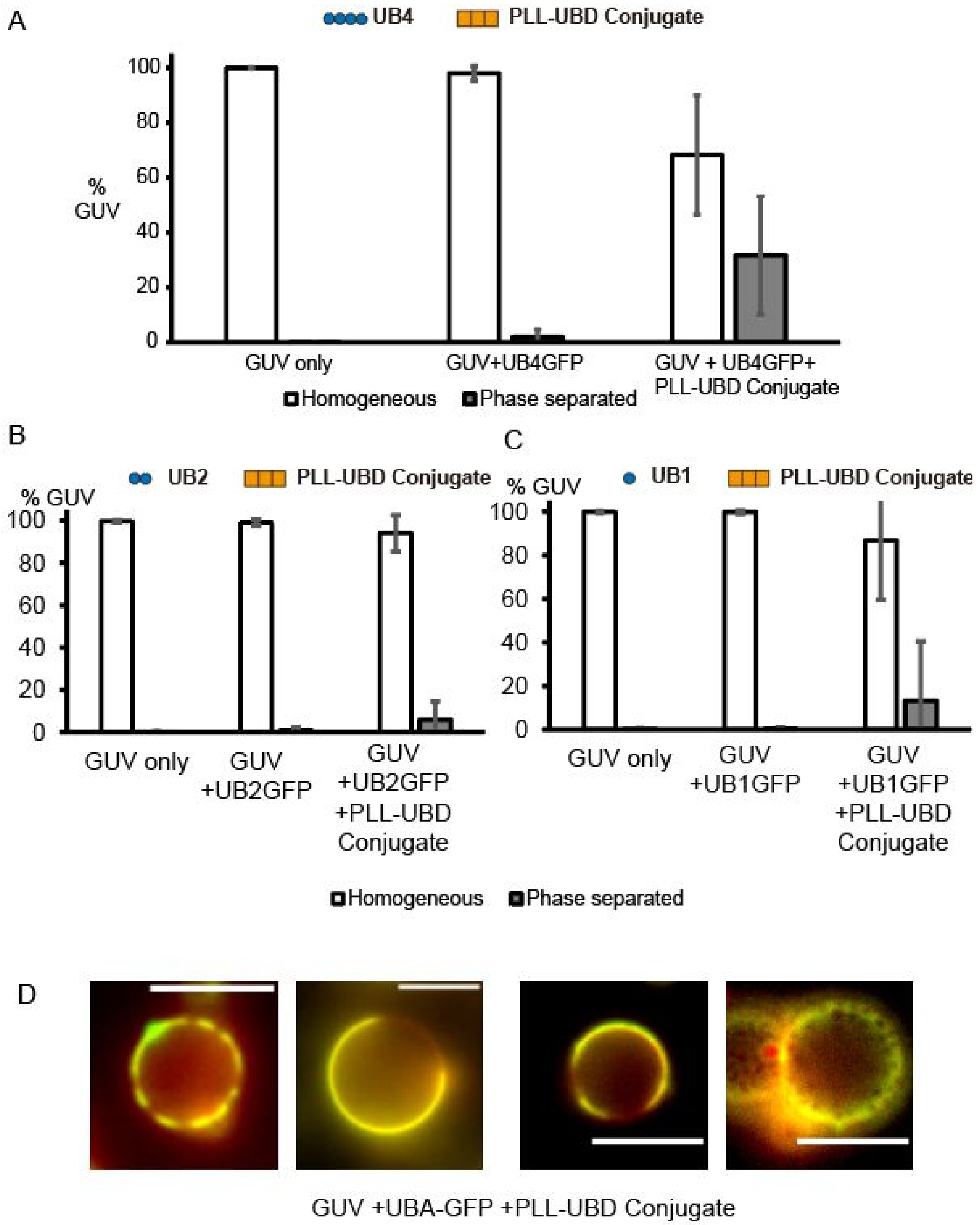

Figure S9. Phase separation of polyUB cargo on the membranes by PLL based UBD Conjugate interaction. The lipid composition was DOPC 35.0%, DPPC 19.8%, cholesterol 35%, Ni-DGS 10%, and TR-DHPE 0.2%, and incubation conditions of the proteins were 4µM UB4-GFP and ~17µM PLL-UBD Conjugate each. (A) Statistical distribution of the resulting phase behavior of GUVs. Statistical analysis from 25 image stacks taken from 3 independent experiments. Error bars represent standard deviations within 25 images analyzed. Each image was analyzed for % of vesicles in each phase state, and the numbers were averaged for the final outcome. (B) UB2-GFP and PLL-UBD Conjugate experiment. (C) UB1-GFP and PLL-UBD Conjugate experiment. (D) Example images showing phase-separated behavior after introducing PLL-UBD Conjugate. Scale bars are 5 µm.

S10. Supplementary figure 10

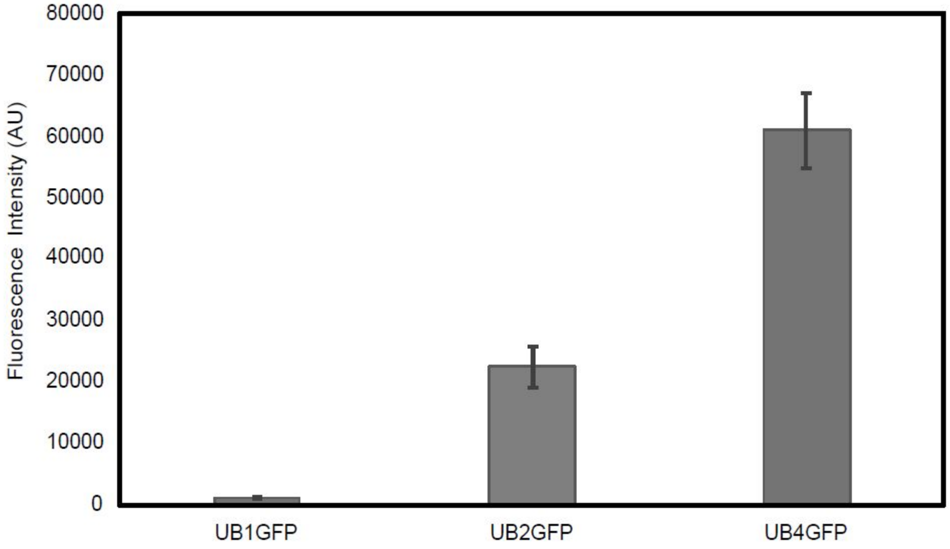

Figure S10. Statistical distribution of the maximum fluorescence intensity of droplet condensates formed by UB proteins and PLL-UBD Conjugate in solution. Peak fluorescence intensity at the middle of each droplet was quantified for comparison. Error bars represent standard deviations of droplet intensity values of at least 50 unique droplets from 3 z-stack images for each UB cargo protein. It is clear that longer UB chain-based droplets are more enriched with the cargo fluorescence.

S11. Supplementary figure 11

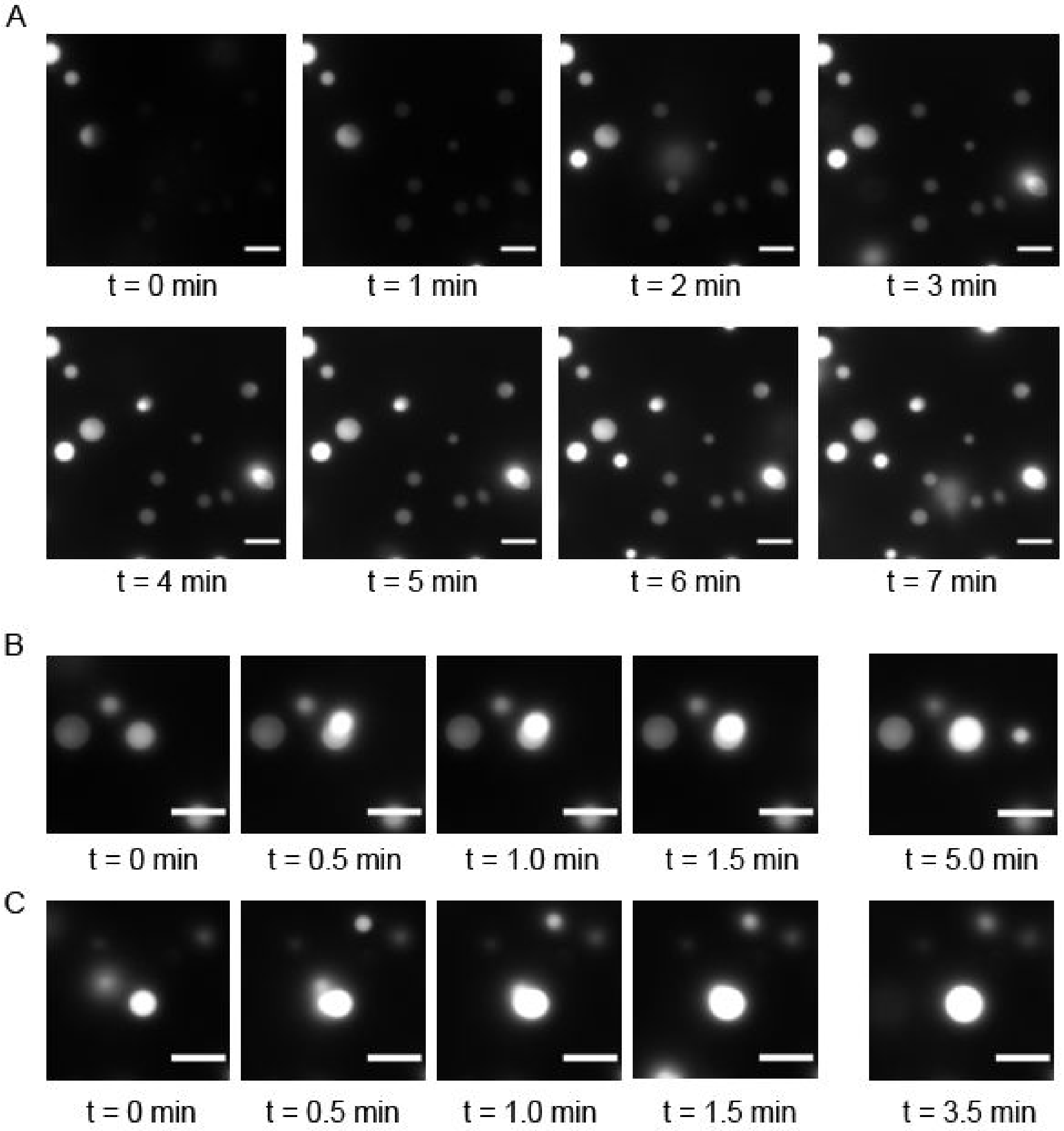

Figure S11. Liquid-like property of the protein droplets formed between UB4-GFP and PLL-UBD Conjugate. (A) Fluorescence recovery after photobleaching experiments on the droplets. GFP fluorescence images were taken. Condensates at the middle of the images were photobleached as a square pattern. As time elapses, their fluorescence intensities recover by fluidic exchange of molecules in solution. A partially bleached condensate recovery is also visible on the left. All images were scaled identically for the brightness and contrast. (B) Example of two condensate droplets coalescing with each other. Two droplets being spontaneously combined into one is evident. (C) Example of multiple condensate droplets coalescing into a single droplet. At 0.5min two droplets coalesced and in 1.0min the third droplet from above also coalesced to eventually merge into a round single droplet at 3.5 min. Coalescing behavior suggests the condensates are fluidic and liquid-like by nature.
